# Grown to be Blue—Antioxidant Properties and Health Effects of Colored Vegetables. Part I: Root Vegetables

**DOI:** 10.3390/antiox8120617

**Published:** 2019-12-04

**Authors:** Spyridon A. Petropoulos, Shirley L. Sampaio, Francesco Di Gioia, Nikos Tzortzakis, Youssef Rouphael, Marios C. Kyriacou, Isabel Ferreira

**Affiliations:** 1 Crop Production and Rural Environment, Department of Agriculture, University of Thessaly, 38446 Nea Ionia, Greece; 2 Centro de Investigação de Montanha (CIMO), Instituto Politécnico de Bragança, Campus de Santa Apolónia, 5300-253 Bragança, Portugal; shirleylsampaio@gmail.com; 3 Department of Plant Science, The Pennsylvania State University, University Park, PA 16802, USA; fxd92@psu.edu; 4 Department of Agricultural Sciences, Biotechnology and Food Science, Cyprus University of Technology, 3603 Limassol, Cyprus; nikolaos.tzortzakis@cut.ac.cy; 5 Department of Agricultural Sciences, University of Naples Federico II, 80055 Portici, Italy; youssef.rouphael@unina.it; 6 Department of Vegetable Crops, Agricultural Research Institute, 1516 Nicosia, Cyprus; m.kyriacou@ari.gov.cy

**Keywords:** anthocyanins, antioxidant activity, beet root, betacyanins, cyanidin, blue potatoes, carotenoids, flavonoids, natural colorants, sweet potato

## Abstract

During the last few decades, the food and beverage industry faced increasing demand for the design of new functional food products free of synthetic compounds and artificial additives. Anthocyanins are widely used as natural colorants in various food products to replenish blue color losses during processing and to add blue color to colorless products, while other compounds such as carotenoids and betalains are considered as good sources of other shades. Root vegetables are well known for their broad palette of colors, and some species, such as black carrot and beet root, are already widely used as sources of natural colorants in the food and drug industry. Ongoing research aims at identifying alternative vegetable sources with diverse functional and structural features imparting beneficial effects onto human health. The current review provides a systematic description of colored root vegetables based on their belowground edible parts, and it highlights species and/or cultivars that present atypical colors, especially those containing pigment compounds responsible for hues of blue color. Finally, the main health effects and antioxidant properties associated with the presence of coloring compounds are presented, as well as the effects that processing treatments may have on chemical composition and coloring compounds in particular.

## 1. Introduction

**Figure 1:**
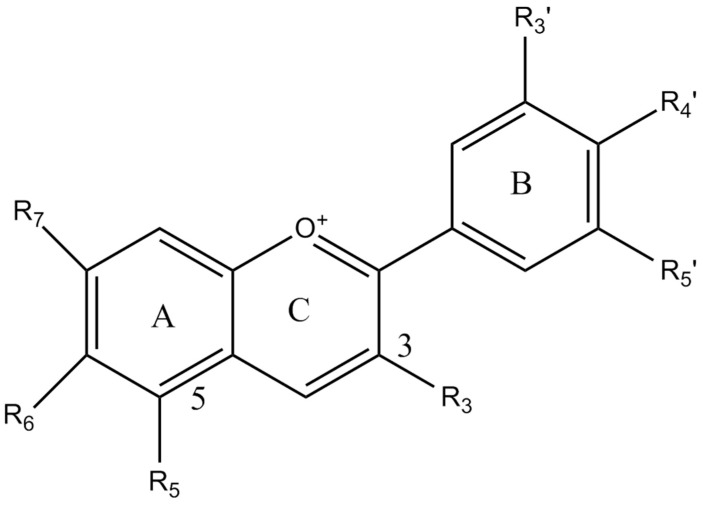
The core structure of anthocyanins with two aromatic benzol rings (A and B rings) and a portion cyclized with oxygen (C ring).

**Figure 2:**
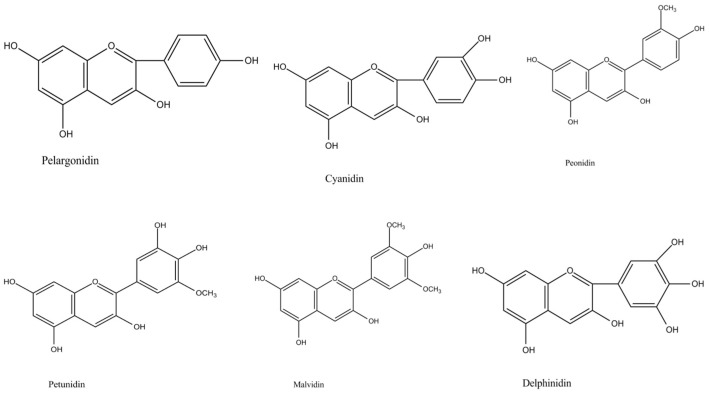
The main anthocyanins detected in root vegetables.

Root vegetables display various colors which usually depend on the presence of three major classes of compounds, namely, flavonoids, betalains, and carotenoids, which they may define their visual appearance and consumer perception [1,2]. Anthocyanins are flavonoids responsible for the different shades of plant epidermal tissues such as purple, blue, red, and pink colors, aiming at attracting pollinators and contributing to the overall plant antioxidant mechanisms under abiotic and biotic stress conditions [3]. They also participate in several physiological processes of the plant, including photosynthesis and plant interactions with the environment [4]. They are produced via the phenylpropanoid pathway and the conversion of leucoanthocyanidins into colored anthocyanidin and glycoside derivatives via anthocyanidin synthase and other enzymes [1,5]. The great number of anthocyanins isolated in nature so far and their high structural variation across plant species raised research interest in these compounds during the last few years in search of novel natural colorants [6,7]. The structural variation of anthocyanins is related to the substitution of hydroxyl and methoxyl groups in the B ring, glycosidic substitution at positions 3 and 5 of the A and C rings, and the possible acylation of glycosidic substitutes with aliphatic and cinnamic acids (Figure 1 and Figure 2) [6]. These structural differences may infer significant variations in the biological activities and antioxidant properties of vegetable products. For example, Oki et al. [8] suggested that antioxidant activities of purple sweet-potato extracts from peonidin-rich cultivars were attributed to anthocyanins, whereas, in those extracts from cyanidin-rich cultivars, the antioxidant capacity was due to the phenolic compounds. Other compounds that transfuse blue color in nature are quinones, quinodes, and various alkaloids which are usually present in fungi, bacteria, and in the animal kingdom [9]. Quinones and quinodes include carbonyl groups within aromatic rings, and they also show a great variation from a structural point of view [9], while alkaloids contain nitrogen atoms and are divided into several distinct classes, including pyridine alkaloids, phenazine alkaloids, and linear tetrapyrrole and indole alkaloids, with different coloring attributes [9].

**Figure 3:**
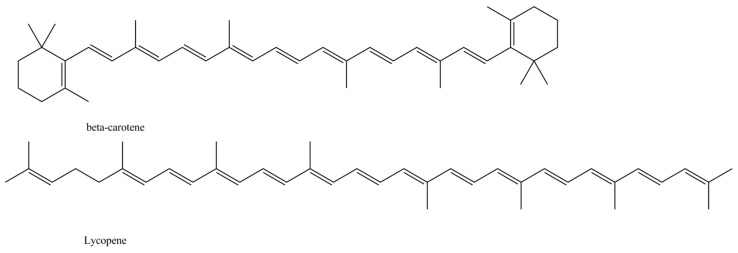
The main carotenoids detected in root vegetables.

**Figure 4:**
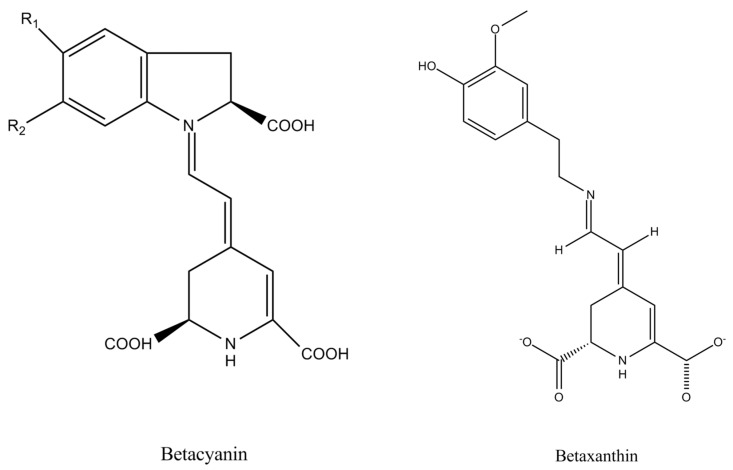
The main betalains detected in root vegetables.

On the other hand, carotenoids are mainly responsible for yellow and orange color with several distinct compounds being detected so far in various vegetables [6,10,11,12], while betalains such as betacyanins and betaxanthins are also important for the violet and yellow pigmentations, respectively [13]. The main detected carotenoids are β-carotene and lycopene, which are unsaturated hydrocarbons, and they differ in terms of the β-rings, where β-carotene molecules have both ends (Figure 3), and they usually present synergistic effects [14]. Both are fat-soluble, and the number of conjugate double bonds in their structure is closely related to their superoxide inhibitory effect [15,16]. Betacyanins and betaxanthins differ in the moiety derived from betalamic acid, as towel as the fact that betaxanthins are produced from the condensation of betalamic acid with amino acids and they never show glycosidation, whereas betacyanins are the result of condensation of betalamic acid with imino compounds (Figure 4) [17,18]. Further differences are observed within each main class of betalains, namely, betacyanins and betaxanthins, with several structures identified resulting in different individual compounds with different absorption and stability capacity [19]. In particular, the various betacyanins are differentiated through the glycosyl groups attached to the *o*-position of the cyclo-dopa moiety [20], while betaxanthins are differentiated through the conjugated moiety of betalamic acid (amino acids or amines) [20]. The main pigments isolated in the various root vegetables are presented in Table 1. 

The first coloring agents used in food products to improve their visual appearance were produced from natural sources; however, the high cost for the production of these coloring agents, the variation in color shades due to the inert variability in natural matrix compositions, and the increasing needs of the market resulted in the use of synthetic compounds originally derived from coal tar and then produced from petroleum and crude oil (e.g., FD&C blue No. 1 and blue No. 2) [9,59]. The consumer concerns about additives and synthetic compounds, amplified by the reports regarding the health risks and the environmental impact associated with these compounds [60,61,62], necessitated the shift to the root food industry dyes; recently, the food and beverage industry is seeking natural coloring agents that could substitute synthetic dyes and coloring additives [63,64]. The colorant content of root vegetable products is associated with various health benefits including the prevention of modern chronic diseases [65,66,67]. However, they are often highly concentrated in the epidermal layers and skin tissues which are commonly discarded during domestic processing or in industrial applications [68,69,70]. For this reason, the research interest in obtaining natural pigments and bioactive compounds from agro-food waste is gaining ground within the context of circular economy and the sustainable use of natural resources [70,71,72,73,74]. There are also several cases where colorants can be found in high concentrations in the flesh due to the presence of pigments in parenchymal cells, increasing the antioxidant capacity and functional value of these products (e.g., potatoes, beets, carrots, and other root vegetables having colored flesh) [3]. Pigment compounds contribute to the overall antioxidant capacity in a dose-dependent and compound-specific manner [75,76,77], although the bioavailability and the absorption mechanisms within human body still need to be addressed [78]. Notwithstanding the genetic background of each species and/or cultivar, color attributes may be modulated by environmental factors such as the light and temperature conditions, through biotic and abiotic elicitors that may affect chemical composition, hormonal signaling, and enzymatic activities. Although not directly exposed to solar radiation, the pigmentation of root vegetables developing belowground may be indirectly modulated by the level and quality of radiation to which the aboveground plant is exposed [79]. In addition to pre-harvest factors, post-harvest conditions and processing methods may have an impact on bioavailability and biostability of natural matrices and coloring compounds [13,80,81]. Anthocyanins in particular are considered a good option as natural coloring agents due to their low toxicity and the wide range of health effects they present [82]. However, the stability and bioavailability of anthocyanins are affected by several factors (chemical structure and concentration, pH of food matrix, temperature, light, presence of co-pigments, enzymes, and metallic ions, among others), which determine the processing method specificity, and which need to be considered before using these compounds as natural coloring agents in the food industry [83,84]. Moreover, the association of structural differences with biostability and bioavailability is further reflected in the biological activities of these compounds, since, for example, acylated forms are less prone to degradation due to pH variations [14,85]. Therefore, although, for some species, there are already defined protocols for the extraction and processing requirements for obtaining natural colorants (e.g., black carrot, beet root colorants) [71,86,87,88], there is still a gap in the literature for other colored vegetables which could prove valuable candidates for yielding coloring agents.

The present review aims to present the main colored root vegetable crops, focusing on cultivars with colors atypical for the species. Special attention is given to blue- and purple-colored vegetables since natural colorants of these shades are less common in nature and are highly sought by the food industry, since blue shades are more difficult to replicate in food and beverages due to the susceptibility of coloring compounds to external factors (e.g., pH of the food matrix, extraction conditions). Furthermore, the main compounds responsible for uncommon colors are presented, as well as their antioxidant capacity and health-promoting effects. Finally, the effects of processing treatments on color stability are addressed. The presented information in this review was obtained from worldwide accepted databases such as Scopus, ScienceDirect, PubMed, Google Scholar, and ResearchGate, using the respective names of the studied species (both common and Latin names) and the additional terms of the main colorants and “health effects” as keywords.

## 2. Main Colored Root Vegetables

### 2.1. Potato

Potato (*Solanum tuberosum* L., Solanaceae) is the third most important food crop in the world, after wheat and rice [89]. In addition to its nutritional and calorific value, potato varieties also offer bioactive compounds with beneficial effects for human health, such as phenolic compounds and carotenoids, among others [12,23]. Several reports highlighted the beneficial effects of antioxidant-rich potatoes against various diseases, such as cardiovascular diseases [90] and various types of cancer [91,92]. Although yellow- and white-fleshed tubers are the most commonly used ones throughout the world, potato has the highest genetic diversity among cultivated species, with approximately 5000 known varieties with broad variability in terms of flesh and skin color [93]. Red- and blue-fleshed potatoes are particularly rich in phenolic compounds, presenting about three times higher amounts of total polyphenolic content than traditional yellow-fleshed tubers, as well as two to three times higher antioxidant activity [12,23,24,94]. 

Acylated forms of anthocyanins were reported to be the main compounds responsible for the red and purple flesh color of potatoes [94]. In particular, the deep-purple color of potato flesh and skin is associated with the presence of petunidin derivatives, although studies on metabolite profiling revealed a genotype- and tissue-specific pattern regarding the anthocyanin composition [22]. Petunidin was the major anthocyanidin compound found both in the flesh and the peel of purple potato varieties studied by Yine et al. [21]. In this study, petunidin accounted for 63–66% of the total anthocyanidin content of purple peel and flesh. The same findings were observed by Kita et al. [23] when studying purple- and red-fleshed potato cultivars, where petunidin-3-*p*-coumaroylrutinoside-5-glucoside was the major anthocyanin compound found in the purple-fleshed varieties Salad Blue (29.31 ± 0.73 mg∙100 g^−1^ dry weight (dw)), Valfi (43.11 ± 0.37 mg∙100 g^−1^ dw), and Blue Congo (36.32 ± 0.33 mg∙100 g^−1^ dw). Similarly, Nemś et al. [24] identified petunidin-2-*p*-coumarylrutinoside-5-glucoside as the major anthocyanin present in the cultivars Salad Blue (28.34 ± 9.30 mg∙100 g^−1^ dw), Valfi (57.77 ± 28.75 mg∙100 g^−1^ dw), and Blue Congo (75.97 ± 12.38 mg∙100 g^−1^ dw). On the other hand, in red-fleshed potatoes, pelargonidin acyl-glycoside derivatives appear as the main anthocyanin compounds. Kita et al. [23] found pelargonidin-3-*p*-coumaroylrutinoside-5-glucoside as the major anthocyanin present in red-fleshed varieties, such as Rosalinde (15.14 ± 0.12 mg∙100 g^−1^ dw), Herbie 26 (44.46 ± 0.23 mg∙100 g^−1^ dw), and Highland Burgundy Red (126.38 ± 0.71 mg∙100 g^−1^ dw). Yin et al. [21] carried out an acid hydrolysis of the anthocyanins, studying the composition of the aglycones (anthocyanidins), reporting pelargonidin as the main anthocyanidin present in the red-fleshed cultivar Red Cloud No. 1, with a concentration of 11.73 ± 0.16 mg∙100 g^−1^ fresh weight (fw), which corresponded to 82% of the total anthocyanidin content. Other anthocyanin compounds were reported in the literature for red- and purple-fleshed potatoes, including delphinidin, cyanidin, peonidin, and malvidin acyl-glycoside derivatives [21,23]. Moreover, the simulation of domestic cooking processing and gastrointestinal digestion of *Solanum tuberosum* L. cv Vitelotte noire extracts revealed significant antimicrobial and anti-proliferative activities against *Bacillus cereus* and *Escherichia coli* in the first case (domestic cooking processes) and colon (Caco-2 and SW48) and breast cancer (MCF7, MDA-MB-231) cell lines in the latter case (gastrointestinal digestion) [95]. 

Carotenoids are fat-soluble pigments that can exert antioxidant properties, and they are also present in colored-flesh potatoes. According to Kotíková et al. [25] who compared the carotenoid content of yellow-fleshed, white-fleshed, purple-fleshed, and red-fleshed potato cultivars, significant differences were observed. Interestingly, yellow potatoes showed a much higher average total carotenoid content (26.22 μg∙g^−1^ dw) in comparison to the red and purple-fleshed cultivars (5.69 μg∙g^−1^ dw), indicating that carotenoid pigments are not highly concentrated in the flesh of purple- and red-fleshed potatoes [25].

Yin et al. [21] investigated 10 colored potato cultivars from China and compared the composition and antioxidant activities of their flesh and peel. The authors found that potato peels were on average 15.34 times richer in anthocyanins than the flesh; the antioxidant activity of the peels extracts was also 5.75 times higher on average than that of the flesh extracts [21]. In the same study, the flesh extracts of cv. Purple Cloud No.1 showed the strongest antioxidant activity among all the tested varieties, along with the highest total content of anthocyanidins (43.38 mg∙100 g^−1^ fw), a correlation which indicates anthocyanins as a major contributor to the antioxidant activity of colored potatoes [21]. 

Recently, there was increasing interest by consumers and food producers in colored potato varieties, due to their attractive organoleptic features (color and taste) and health-promoting chemical composition [12]. The increasing interest of the market for colored potato is stimulating private and public breeding programs to release new specialty potato cultivars such as the red-skin and red-flesh TerraRossa and AmaRosa or the purple-skin yellow-flesh cultivar Huckleberry Gold and Peter Wilcox, marketed as “Purple Sun” or “Blue Gold”, which are also characterized by a higher content of anthocyanins, anthocyanidins, and other phenolic compounds [96]. The consumption of anthocyanin-rich food products such as purple-flesh potatoes is associated with the modification of the expression of various genes involved in the metabolism of lipids, inflammation, and energy homeostasis in liver and/or fat tissues [97,98]. Moreover, extracts from purple potato tubers may improve the differentiation of gut epithelia and its barrier function against gut epithelial inflammation through the activation of AMP-activated protein kinase (AMPK) and the increase of CDX2 gene [99]. Color-fleshed potatoes are an excellent source of bioactive compounds that are effective against human colon cancer cell lines (HCT-116 and HT-29); however, prolonged storage may affect their antiproliferative and pro-apoptotic activities [100]. Red- and purple-fleshed potato extracts were also effective against *tert*-butyl hydroperoxide (*t*-BHP)-induced hepatotoxicity through the recovery of serum alanine aminotransferase (ALT) and aspartate aminotransferase (AST) enzyme activities [101]. Therefore, a market niche for colored potato-based food products was created, such as potato chips and crisps. However, the frying process to produce colored potato crisps can cause a 38–70% degradation of anthocyanin compounds, with pelargonidin and malvidin acyl-glycoside derivatives being more stable during the frying process in comparison to petunidin acyl-glycoside derivatives [23]. Nevertheless, despite the reduced contents of anthocyanins in processed compared to raw potatoes, colored potato crisps can present bright attractive colors, in addition to 2–3 times higher antioxidant activities and 40% higher contents of polyphenols than standard snacks made of commonly used yellow potatoes and corn [23,24]. Moreover, in a recent study, Nemś and Pęksa [94] incorporated dried red- and purple-fleshed potatoes into fried snacks and doughs, reporting a beneficial effect on the inhibition of oxidative changes in lipids compared to control material (yellow snacks), particularly when incorporating material from purple-fleshed potato varieties of Blue Congo and Valfi. These effects were attributed to the higher content of colored snacks in polyphenols and anthocyanins than control, with petunidin 2-*p*-coumaroyl-rutinoside-5-glucoside being the major anthocyanin present in both cultivars [94]. Other domestic cooking processes such as boiling, baking, steaming, and microwaving may also affect the anthocyanin content and antioxidant capacity of colored potatoes, with processing (steaming and microwaving) showing the best results in retaining anthocyanin content and antioxidant activity [102,103,104,105,106]. Thermal processing affects not only anthocyanins but also carotenoids which are heat-sensitive and may be degraded, isomerized, or oxidized after domestic cooking processes [25]. According to Qiu et al. [107], anthocyanin content decreased with prolonged drying time and high drying temperatures due to higher degradation rates and shorter half-life values compared to shorter drying procedures with lower temperatures. Therefore, the antioxidant properties of colored potatoes can be beneficial not only to human health but also to the shelf life of processed food products. Another important aspect of processed food products based on processed colored potatoes is that the various types of processing (French fries, chips, and puree) reduce the content of antinutritional factors such as the glykoalkaloids α-chaconine and α-solanine, thus increasing the overall nutritional quality of the semi-processed and final products [108].

### 2.2. Sweet Potato

Sweet potato (*Ipomoea batatas* (L.) Lam., Convolvulaceae) is a perennial species native to Latin America which is highly appreciated for its fleshly tuberous roots that are widely used in the food and non-food industry depending on starch content and properties [109,110]. In Japan, purple sweet-potato anthocyanins are used as ingredients in several food products and beverages [111,112]. The flesh of the roots is usually white, yellow, or orange, although several cultivars with purple-colored flesh and a high content of anthocyanins also exist [113,114]. It is the fourth most produced vegetable in the world after potato, cassava, and tomato with a total production of 113 million tons in 2017, most of which (63.8%) were produced in China [115]. The nutritional value of the edible roots consists in the richness of carbohydrates, dietary fibers, vitamins, and minerals, while several polyphenolic compounds, peptides, and carotenoids are also present in considerable amounts in the flesh [116] and peels [74] of the tubers. The high calorific value of sweet potato roots makes the species one of the most important food crops in terms of calorific contributors to the human diet [117]. Starch is the main calorific component of sweet-potato tubers with significant variation in its structural and functional properties which depend mostly on the genotype and are not correlated with flesh color [118], although, using a proteomic approach, a recent study revealed that starch degradation may contribute to anthocyanin biosynthesis and accumulation in purple sweet-potato roots [119]. Chlorogenic acid, protocatechuic acid, salicylic acid, and caffeoylquinic acid derivatives are the main phenolic acids detected in purple sweet-potato roots and are responsible for their antioxidant capacity [48,120,121], while orange-fleshed sweet-potato cultivars are rich in provitamin A and also show significant antioxidant activity [113,122,123]. Moreover, in the study of Lebot et al. [124], the antioxidant activity of sweet-potato cultivars with purple, orange, and white flesh was correlated mostly with the presence of caffeoylquinic acid derivatives and less with total anthocyanin content, whereas, according to Oki et al. [8], the contribution of phenolic compounds in radical-scavenging activity is also dependent on the genotype. In contrast, according to the study of Kubow et al. [125], anthocyanins are responsible for the antioxidant capacity of sweet-potato tubers. In the same study, it was reported that the anthocyanin species were detected in the small intestinal and the ascending colonic vessel, depending on the sweet-potato genotype, and the antioxidant activity was increased accordingly [125]. According to the report of Meng et al. [126] who studied the digestion kinetics of sweet-potato polyphenols, the maximum release was recorded 2 h after intestinal digestion and was induced by gastric acid and pepsin [126]. Moreover, acylated anthocyanins from sweet potato are considered as complex and less susceptible to intestinal degradation [127,128], while Sun et al. [129] suggested a prebiotic-like activity of anthocyanins through the modulation of microbiota in the intestine. These results highlight the importance of unraveling the bioavailability and bioaccessibility patterns influencing the antioxidant potential of purple-fleshed sweet potatoes [125].

Acylated anthocyanins are responsible for the intense color of purple-fleshed sweet potatoes [66,130], which renders them good candidates sources for natural colorants with practical application in the food industry [131]. Moreover, peels are also a good source of natural pigments since they contain significant amounts of anthocyanins, and the exploitation of this by-product for obtaining coloring agents would increase the added value of the sweet-potato crop [74]. The total anthocyanin content and compositional profile may differ among the various genotypes, with a total of 39 different anthocyanins isolated so far [132,133]. The main anthocyanins isolated from purple sweet-potato extracts were identified as cyanidin, peonidin, and pelargonidin derivatives [26,27,28,29,30,110,134,135], which were effective against alcohol-induced liver injury in rats when administrated at median doses (100 mg∙kg^−1^ body weight), whereas higher doses (300 mg∙kg^−1^ body weight) had a pro-oxidant effect and promoted liver injury [136]. Moreover, cyanidin 3-caffeoyl-*p*-hydroxybenzoyl sophoroside-5-glucoside which was isolated from purple-fleshed sweet potatoes was shown to be effective both in vitro and in vivo in inhibiting hepatic glucose secretion and reducing blood glucose [137,138,139], while peonidin suppressed the excessive expression of the HER2 protein showing anticancer activities [140]. According to Luo et al. [141], cyanidin 3-caffeoyl-feruloyl sophoroside-5-glucoside and peonidin 3-dicaffeoyl sophoroside-5-glucoside were the most effective anthocyanins isolated from the purple sweet-potato cultivar Eshu No. 8. In another study, the oral administration of purple sweet-potato color attenuated cognitive deficits in domoic acid-treated mice through mitochondrial biogenesis signaling and the decrease of p47phox and gp91phox expression [142], while similar results were reported by Zhuang et al. [143], who suggested the regulation of AMPK/autophagy signaling as the mechanism of action. The same pigment was effective against neuroinflammation in mouse brain through the inhibition of mitogen-activated protein kinase (MAPK) and the activation of nuclear factor κB (NF-κB) [144], as well as against bladder cancer through the inhibition of the signaling of phosphatidylinositol-4,5-bisphosphate 3-kinase/Akt or protein kinase B (PI3K/Akt) [145]. In particular, for mitochondrial biogenesis, it was reported that anthocyanins can bind and stimulate estrogen receptor-α and then increase the expression of nuclear respiratory factor-1 (NRF-1) [146]. Anthocyanin-rich extracts from purple sweet potato were moderately effective against human colon cancer cell lines (HCT-116 and HT-29) through the inhibition of tyrosine kinase activity, whereas they showed no effectiveness against the CCD-33Co cell line [67]. Moreover, Yoshimoto et al. reported that the antimutagenic activity of sweet-potato extracts was attributed mainly to cyanidin content (63% inhibition of mutagenicity of Trp-1 against *Salmonella tymphimurium* TA 98 at the dose of 1.5 mM) [147], while Zhao et al. suggested that anthocyanin-rich extracts from sweet potato are potent anti-aging (at the dose of 1000 mg/kg body weight), anti-hyperglycemic (at the dose of 1 g/kg body weight), and anti-tumor agents (68% tumor inhibition at the dose of 1000 mg/kg body weight) [148]. In another study, highly acylated anthocyanins showed effectiveness against hyperuricemia and kidney inflammation in allopurinol-induced hyperuricemic mice [149], while purple sweet-potato color reduced renal damage through the downregulation of vascular endothelial growth factor receptor (VEGFR2) expression [150]. The regular intake of anthocyanins is also highly associated with the prevention of various chronic liver diseases, and it can reduce lipid accumulation in liver tissues and alleviate oxidative stress and hepatic inflammation [25,102,151,152,153,154,155,156]. Other hepatoprotective effects of purple sweet potatoes include hepatic insulin resistance in high-fat diet-treated mice through the decrease of reactive oxygen species (ROS) production and the inhibition of endoplasmic reticulum (ER) liver stress (administration of purple sweet-potato color at the dose of 700 mg/kg/day) [157], through the decrease in the expression of ionized calcium-binding adapter molecule 1 (Iba1), tumor necrosis factor-α, interleukin-1β, suppressors of cytokine signaling3 (SOCS3), and galectin-3 (administration of purple sweet potato color at the dose of 500 mg/kg/day) [158], or through the inhibition of nucleotide-binding domain, leucine-rich repeat (NLR) family, pyrin domain containing 3 (NLRP3) inflammasome activation (administration of purple sweet potato color at the dose of 700 mg/kg/day) [159]. Moreover, the combinative use of black soybean and purple sweet potato (mixtures of 2:2 for black soybean and purple sweet potato) resulted in improved insulin sensitivity in streptozotocin-induced diabetic rats through the improvement of insulin and insulin receptor substrate-1 (IRS-1) expression, the increase of superoxide dismutase (SOD) levels, and reduced pancreatic necrosis [160]. In a similar study, the mixture of *Curcuma longa* L. and sweet potato (at the dose of 2–5 mg/kg body weight) showed significant immunomodulating properties in murine leukemia retrovirus-infected mice [161]. The administration of purple sweet potato to obese mice fed with a high-fat diet exhibited anti-obesity effects and attenuated gain weight [162]. Other bioactive compounds of purple sweet potatoes include alkali-soluble polysaccharides which presented anti-inflammatory properties in lipopolysaccharide (LPS)-treated macrophages (RAW 264.7) through the inhibition of nitric oxide, interleukin (IL)-6, IL-1β, and tumor necrosis factor alpha (TNF-α) and the increase of IL-10 [163], as well as anti-inflammatory effects against intestinal inflammation on dextran sulfate sodium (DSS)-induced mice [164], hepatoprotective properties [165], and immunomodulatory effects [166,167,168]. Non-flavonoid compounds and kaempferol derivatives are also present in sweet-potato tuber tissues, and they contribute to the overall bioactive capacity of sweet potato [28].

Processing and storage conditions are important for the chemical composition and the visual quality of sweet-potato tubers, with heating treatments and higher pH having a detrimental effect on anthocyanins and starch content and on flesh color [139,169,170,171,172]. Pretreatments such as blanching, osmotic dehydration, ultrasound-assisted dehydration, and ultrasound-assisted osmotic dehydration before microwave drying also had an impact on total phenolic and anthocyanin content of orange- and purple-fleshed sweet-potato slices [173]. Domestic cooking processes may also affect total anthocyanin and total phenolic content, with steaming suggested as the mildest process to retain the highest amount of total anthocyanins compared to fresh samples, while, at the same time, an increase in total phenolic content was observed by Phan et al. [174]. In a similar study, steaming, roasting, and boiling were suggested as the best cooking methods for retaining total phenolic, anthocyanin, and carotenoid content, respectively, in white, yellow, orange, light-purple, and deep-purple sweet-potato tubers [10]. 

### 2.3. Carrot

Carrot (*Daucus carota* L. ssp. *sativus* Hoffm.) belongs to the Apiaceae family and is a highly appreciated vegetable consumed for its edible fleshy roots. There are two cultivar groups depending on root color, namely, the carotene or western carrot (*Daucus carota* ssp. *sativus* var. *sativus*) and the eastern or anthocyanin carrot (*Daucus carota* ssp. *sativus* var. *atrorubens* Alef.), which are widely cultivated throughout the world with an annual production of 42.8 million tons including turnips [115]. Although the orange-colored carrots are the most popular ones, a broad genetic basis exists with many other shades of root flesh (red, white, yellow, black, purple, or multi-color) which attract interest due to their nutritional value and associated health effects [175]. Recently, new genetically biofortified cultivars were developed which contain not only α- and β-carotene but also anthocyanins and lycopene [176]. In particular, for black or purple carrots, several research reports highlighted their beneficial health effects on human health, and they are widely used so far as natural sources of blue color and functional ingredients in the food industry [33,40]. 

Black carrots contain high amounts of mono-acylated anthocyanins which are less prone to thermal degradation, while they can retain their color at various pH values and storage conditions [31,32,34]. These functional and structural characteristics of colored carrot pigments make them good candidates for the extraction of natural colorant agents with practical applications in the food industry, especially in food products with low pH, in beverages and confectioneries [40,131,177]. However, despite their stability under various conditions, Espinosa-Acosta et al. [82] did not suggest their use in food models such as yoghurt and jelly, except for the case of ethanolic extracts of black carrots, which could be incorporated into jellies to increase the antioxidant activity of the final product. Moreover, Assous et al. [86] suggested the use of black-carrot pigments as coloring agents in hard candy and sweet jelly without significant differences in the sensorial profile compared to the control, while the same pigments protected sunflower oil from lipid peroxidation. The use of black-carrot extracts was also proposed for the preparation of jams and marmalades, where the main anthocyanins were slightly affected after gastric ingestion and storage at 4 °C [178], as well in co-pigmentation with other natural colorants (e.g., plum, jamun, strawberry, and pomegranate juices), to increase the color stability to heat treatments and pH variation [179]. On the other hand, red and yellow carrots are rich in carotenoids and lycopene and β-carotene in particular [37,38], which, according to Horvitz et al. [180], are both bioavailable and can provide a significant amount of these carotenoids to human diet. 

The main anthocyanins detected are mostly cyanidin derivatives, and, according to Frond et al. [48], the most abundant anthocyanin identified in black-carrot extracts was cyanidin-3-(*p*-coumaroyl)-diglucoside-5-glucoside. In the study of Montilla et al. [33], the main detected anthocyanins in *Daucus carota* subsp. *sativus* var. *atrorubens* Alef. were identified as acylated cyanidin 3-xylosyl(glucosyl)galactosides with sinapic acid, ferulic acid, and coumaric acid, and significant differences were observed between genotypes (Antonina, Beta Sweet, Deep Purple, and Purple Haze) in terms of total and individual anthocyanin content. Similar results were reported in the earlier study of Kammerer et al. [181], with acylated and non-acylated cyanidin derivatives found in the highest amounts, while they also suggested significant differences between 15 different black-carrot cultivars, as well as between roots of the same cultivar. Moreover, in black-carrot juice, two more compounds were identified, namely, cyanidin-3-xylosyl-galactoside and cyanidin-3-xylosyl (feruloylglucosyl)galactoside [35], while Schwarz et al. [36] isolated four more pigments identified as vinylphenol and vinylguaiacol adducts of cyanidin derivatives which are formed during the storage of juice through the reaction of phenolic acids with anthocyanins. Regarding the health effects of anthocyanins, extracts from purple carrot were moderately effective against HCT-116 human colon cancer cell lines through the inhibition of tyrosine kinase activity, whereas they showed no effectiveness against HT-29 and CCD-33Co cell lines [67]. Yet, black-carrot crude extracts exhibited significant antioxidant, cytoprotective, and anti-angiogenic properties, indicating a synergistic effect of the various polyphenols (anthocyanins, phenolic acids, and flavonoids) contained in the root extracts [182]. Although non-digested purple carrot extract is more potent than the digested extract, Olejnik et al. [183] reported that gastrointestinal digested purple-carrot extract had intracellular ROS-inhibitory activity and protected colonic cells against oxidative stress by reducing oxidative DNA damage by 20.7%. According to Blando et al. [184], the anthocyanin-rich extracts from black carrots contained mostly anthocyanins acylated with cinnamic-acid derivatives, which exhibited anti-inflammatory activities through the reduction of the expression of endothelial inflammatory antigens. Apart from anthocyanins, black carrots are a good source of phenolic acids, namely, chlorogenic and caffeic acids, which contribute to the overall antioxidant capacity [48,185]. 

Processing may affect the chemical composition and antioxidant properties of black-carrot juice, and the use of pectinase during maceration increased the total anthocyanin content, the overall antioxidant capacity, and the juice yield of enzyme-treated compared to normally pressed juice [186,187]. The use of ultrasound and mild heating (50 °C) may increase the extraction yield of anthocyanins from black-carrot pomace, especially the content of cyanidin-3-xyloside-galactoside-glucoside-ferrulic acid and cyanidin-3-xyloside-galactoside, which were detected in the highest amounts [71]. Another processing treatment which could increase the bioavailability and stability of anthocyanins in black-carrot-based food products is the microencapsulation of anthocyanin-rich extracts [83]. Moreover, wounding stress may increase anthocyanin content, chlorogenic acid in particular, thus improving the nutritional and functional value of the obtained food products [188].

### 2.4. Beet Root

Beet or table beet (*Beta vulgaris* L.) belongs to the Amaranthaceae family and is commonly used for its edible fleshy red roots and tender leaves. Beet roots contain betalains, a class of compounds which is further divided into betacyanins and betaxanthins [39]. The composition of betalains and the ratio of betacyanins to betaxanthins depends on tyrosine production and its conversion to betalains, with significant differences observed between red and yellow beet roots [189]. The most commonly found betacyanins are betanins which are responsible for the red vivid color of beet roots, and they are water-soluble and sensitive to prolonged heating [40]. Betanins are commercially available as color additives (E162) in powder form or as juice concentrates following vacuum evaporation [39]; however, there is a great diversity in flesh color among the beet-root genotypes with variable intensities of red color or other shades ranging from white to orange. Apart from the genotype, color intensity is also affected by growing conditions and maturity stage, storage conditions, and processing treatments [88,190,191]. Beet roots with yellow color are most abundant in betaxanthins, while betacyanins are present in lesser amounts [41]. In the study of Wettasinghe et al. [192], beet-root genotypes with diverse flesh colors exhibited significant differences in antioxidant activity and in phase II enzyme induction capacity, which is associated with cancer chemoprotective effects [192]. Moreover, Lee et al. [42] reported that betanine and betaine extracted from red- and yellow-colored beet roots were effective against HepG2 cell proliferation in a dose-dependent manner. In the same study, the main identified betalains detected in the cultivar with yellow roots (Burpee’s Golden Globe) were vulgaxanthin I and betanin [42]. Vulić et al. [193] also reported that the beet-root pomace, a by-product of the beet-root juice extraction, has a high content of betalains and phenolic compounds which exhibited in vitro antiradical activities against 2,2-diphenyl-1-picrylhydrazyl (DPPH) radicals and in vivo antioxidant and hepatoprotective activity, suggesting that it could be used as an excellent nutraceutical resource or an ingredient of functional food products.

### 2.5. Yam

Yam includes various species of the *Dioscorea* genus (Dioscoreaceae), although sometimes it is confused with other root vegetables such as sweet potatoes, oca, taro, etc., which locally may be referred to as yams [194]. Tuber flesh color can be white, yellow, red, or purple depending on the cultivar, with significant differences in bioactive compound content and antioxidant properties [194,195]. Purple yam or water yam (*Dioscorea alata purpurea*) is usually cultivated in tropical and subtropical regions of the world, and its edible roots are very rich in starch and amylose [196], although a great variation in chemical composition of the edible parts of the species was reported [197]. Resistant starch from purple yam (*D. alata*) was effective against hyperlipidemia in high-fat diet-fed hamsters through the amelioration of lipid metabolism and the modulation of gut microbiota [196,198]. Moreover, extracts from roots significantly reduced blood glucose levels in Wistar rats with alloxan-induced hyperglycemia [199] or cholesterol (total and low-density lipoprotein (LDL)) and triglycerides in hypercholesterolemic rats [200], ameliorated doxorubicin (DOX)-induced cardiotoxicity [201], showed protective effects against aniline-induced spleen toxicity [202] and in vivo anti-inflammatory activities against λ-carrageenan-induced paw edema in mice [203], and could be used as an adjuvant in bone-marrow-derived dendritic cell (DC)-based vaccines for cancer therapy [204]. *D. alata* root extracts may also alleviate cellular fibrosis through the downregulation of the transforming growth factor-beta (TGF-β)/Smad signaling pathway and the modulation of epithelial–mesenchymal transition (EMT) expression in kidneys [205]. On the other hand, according to Chan et al. [206], root extracts are also effective against CCl_4_-induced liver injury and hepatic fibrosis. Other health effects include the improvement in function of large bowel and modulation of fecal microflora [207], beneficial effects in gastrointestinal function [208] and cognitive ability [209,210], and the activation of the immune system [211]. The root color of purple yam (*D. alata*) is attributed to the high content of anthocyanins which exhibit significant antibacterial activities [212], anti-inflammatory effects on trinitrobenzenesulfonic acid (TNBS)-induced colitis in mice [213], antiglycative properties [214], and antidiabetic properties [215,216]. The main detected anthocyanins in this species were identified as cyanidin, pelargonidin, and peonidin-type compounds and alatanins A–C [43]; however, the individual compound profile and the overall anthocyanin content are affected by maturity stage and the expression of the concomitant genes [44]. Apart from *D. alata*, which is considered the main purple yam, there are also cultivars of *D. trifida* or cush-cush yam which contain peonidin, cyanidin, and pelargonidin aglycones [45]. Other compounds with bioactive properties are also present, namely, phenolic acids such as ferulic, sinapic, vanillic, caffeic acid, and *p*-coumaric acid, and others, which presented immunomodulatory properties [217,218], proteins with estrogen-stimulating activities that may relieve menopausal syndrome [219], allantoin and dioscin [220], dioscorin [221], or β-sitosterol and ethyl linoleate with anti-atherosclerotic activity [222]. On the other hand, carotenoids and β-carotene in particular are responsible for the root color of yellow yam (*D. cayennensis*) [47]. Yam roots may contain antinutritional factors such as tannins and diosgenin, which also present bioactive properties. For example, antidyslipidemic effects were reported for diosgenin extracts from purple and yellow yams without affecting body weight gain [220,223], while diosgenin and furostanol glycosides and spirostanol glycosides were effective against the proliferation of various cancer cell lines (MCF-7, A 549, and Hep G2) [224].

A very common use of purple yam is the substitution of wheat flour for bakery products and food products in general without affecting the sensorial acceptance of the products by consumers [225,226,227], while yam flour can be used for gluten-free bakery products [228].

### 2.6. Onion

Onion (*Allium cepa* L., Alliaceae) is one of the most important species of the *Allium* genus, which is commonly used for its edible bulbs. There is a great number of cultivars available with a great diversity in color, which usually refers to bulb skin color, since, in most cases, the presence of pigments is limited to the outer skins of the bulb [229]. In many countries, onion bulbs are considered the main dietary source of flavonoids, a high proportion of which is attributed to the anthocyanin content [70,230]. However, most of the studies refer to red-onion cultivars which contain various polyphenols including acylated and non-acylated cyanidin glucosides, and less information is available about the profile of anthocyanins in purple onions [48,49]. The biosynthesis of anthocyanins involves the shikimate pathway and the activity of anthocyanidin synthase, which catalyzes the production of anthocyanidins, and, after further enzymatic reactions, the various anthocyanins are produced [5]. Comparing green, yellow, red, and purple onion, Benkeblia [231] observed higher total phenolic content and antioxidant properties in red and purple onion-bulb extracts. Similar results were reported by Zhang et al. [232] in a study comparing white, yellow, and red onion, with the latter showing considerably higher total anthocyanin, flavonoid, and polyphenol content, which was also correlated to high antioxidant activity measured through DPPH, 2,2’-azino-bis(3-ethylbenzothiazoline-6-sulfonic acid (ABTS•^+^), and fluorescence recovery after photobleaching (FRAP) assays. Bulb extracts are potent bioactive natural matrices, and, according to the study of Oboh et al. [233], extracts of purple onion were effective against angiotensin-converting enzyme, α-amylase, and α-glucosidase activity, showing significant antidiabetic and anti-hypertensive effects. Moreover, skins of pearl onion exhibited significant anti-inflammatory properties and inhibitory effects against radical-induced DNA scission [234]. In terms of antioxidant activity, purple onions exhibited higher oxygen radical absorbance capacity (ORAC) values than white onions, which indicates a higher concentration of bioactive compounds [235]. A preliminary study conducted by Khiari et al. [236] suggested that, depending on the quality of the plant residues, onion solid waste, also constituted primarily by the outer dry layers of the bulbs, may be used to extract polyphenols with potential antioxidant activity, and the yield of total polyphenols can be optimized using ethanol extracts, with extraction time up to 6 h, while maintaining relatively low extraction temperature (40 °C gave better results than 60 °C).

### 2.7. Other Root Vegetables

Radish (*Raphanus sativus* L., Brassicaceae) is a cruciferous species, well known for its normally white edible fleshy hypocotyls which come in different shapes, sizes, and skin colors. Apart from white-fleshed cultivars, there are also genotypes with pink and purple hypocotyls due to the presence of pigments in the xylem [50]. Pigmentation may also change with the hypocotyl development stage [237]. Purple color implies the presence of anthocyanins, and, according to the study of Reference [51], 60 different compounds were detected and identified as cyanidin glucosides. Most of the anthocyanins are present in acylated forms of cyanidin glucosides which increase their stability, and they could be easily used as natural colorants in functional foods [7,50,238,239], while root extracts may also exhibit beneficial health effects against gastric injuries [240]. 

Purple kohlrabi (*Brassica oleracea* var. *gongylodes*) is another species of the Brassicaceae family with intense purple color, whose edible part is the swollen fleshy meristem. The pigments are located in the meristem skin and consist of cyanidin and cyanidin glucosides which are responsible for the strong antioxidant properties of the species [54,55,56,57]. Examining the antioxidant activity of kohlrabi ethanol and water extracts, Pak et al. [241] observed strong DPPH radical-scavenging activity, and purple kohlrabi extracts had higher antioxidant capacity compared to green kohlrabi extracts. Similarly, comparing green and red kohlrabi, Jung et al. [242] observed that the latter had double the total phenolic content, as well as a higher antioxidant (DPPH, ABTS, and peroxynitrite scavenging activity assay (ONOO^−^)) effect compared to green kohlrabi. In the same study, red kohlrabi methanol extract had stronger anti-inflammatory, antidiabetic, and antioxidant effects than the green kohlrabi methanol extract.

Taro (*Colocasia esculenta* L.) is a root vegetable of the Araceae family with great genetic diversity in plant morphology, including the color of corm flesh, which can be white, purple, brown, or blackish [57,243,244]. The main detected anthocyanins were identified as cyanidin and pelargonidin glucosides, and they exhibit significant antioxidant and anti-inflammatory activities [58].

## 3. Conclusions

Root vegetables with intense and uncommon colors are very important in the human diet, not only because they increase the overall intake of health-promoting compounds, but also because they diversify the daily diet in terms of color, flavor, and chemical composition, which imparts distinct functional effects on the human body. The inclusion of such root vegetables either raw in fresh salads or in cooked dishes may increase palatability and appeal for healthier food products, although proper marketing is always an issue since consumers are usually reluctant to introduce new flavors and unconventional products that break the mold. Nevertheless, the current trends in the food and beverage market and the increased public demand for substituting synthetic compounds with natural alternatives could boost the establishment of these species and help the crossing over from niche products to widely accepted ones with diverse uses in the food industry. Further research is also needed in order to (i) identify those correlations and mechanisms of action responsible for the antioxidant properties and health effects of the pigmented vegetables, (ii) evaluate agronomic practices that will increase the bioactive capacity of the final products through the improved pigmentation, (iii) study post-harvest and processing treatments that will make these compounds less prone to degradation and easier to use in the design of functional foods and as natural coloring agents, and (iv) define efficient extraction protocols that will allow high yields and high stability and quality of coloring agents extracted from plant sources. Finally, increasing the knowledge about the chemical composition and the health effects of individual compounds of colored root vegetables could be further exploited through breeding programs for the production of elite genotypes with increased content of coloring compounds and tailor-made health effects, as well as through plant in vitro strategies for the production of specific natural secondary metabolites and further use in the pharmaceutical and the food and beverage industries. 

## Figures and Tables

**Table 1:** The main pigments isolated in various root vegetables.

Species	Edible Part	Color	Class of Compounds	Compounds	References
Potato (*Solanum tuberosum* L.)	Tuber (stem tuber)	Purple	Petunidin derivatives	Petunidin-3-*p*-coumaroylrutinoside-5-glucoside, petunidin-2-*p*-coumarylrutinoside-5-glucoside	[21,22,23,24]
		Red	Pelargonidin, delphinidin, cyanidin, peonidin, and malvidin acyl-glycoside derivatives	Pelargonidin-3-*p*-coumaroylrutinoside-5-glucoside	[21,23]
		Purple/red	Carotenoids	Neoxanthin, violaxanthin	[25]
		Yellow	Carotenoids	Antheraxanthin	[25]
Sweet potato *(Ipomoea batatas* L. Lam.)	Tuberous root (root tuber)	Purple	Acylated anthocyanins	Cyanidin, peonidin, and pelargonidin derivatives	[26,27,28,29,30]
Carrot (*Daucus carota* L. ssp. *sativus* Hoffm.)	Taproot (swollen hypocotyl and root)	Purple or black	Cyanidin derivatives	Acylated cyanidin 3-xylosyl(glucosyl)galactosides with sinapic acid, ferulic acid, and coumaric acid;	[31,32,33,34,35]
				Vinylphenol and vinylguaiacol adducts of cyanidin derivatives	[36]
		Red and yellow	Carotenoids	Lycopene and β-carotene	[37,38]
Beet root (*Beta vulgaris* L.)	Root (swollen hypocotyl and root)	Purple	Betalains	Betacyanins	[39,40]
		Yellow	Betalains	Betaxanthins	[41]
				Vulgaxanthin I and betanin	[42]
Yam (*Dioscurea* sp. L.)	Tuber (stem tuber)	Purple	Cyanidin, pelargonidin, and peonidin-type compounds; alatanins A–C	Cyanidin 3-hexoside acylated with two hydroxycinnamic acids, cyanidin 3-glycoside acylated with one hydroxycinnamic acid, cyanidin 3-glycoside acylated with one hydroxycinnamic acid, peonidin 3-glycoside acylated with one hydroxycinnamic acid, alatanin-C	[43,44,45,46]
		Yellow	Carotenoids	β-Carotene	[47]
Onion (*Allium cepa* L.)	Bulb (swollen basis of leaves)	Purple	Flavonols and acylated and non-acylated cyanidin glucosides	Dihydroflavonol taxifolin and its 3-, 7-, and 4′-glucosides	[48,49]
Radish (*Raphanus sativus* L.)	Taproot (swollen root and hypocotyl)	Purple	Cyanidin glucosides	Cyanidin 3-(glucosylacyl)acylsophoroside-5-diglucosides, cyanidin 3-sophoroside-5-diglucosides, cyanidin 3-sophoroside-5-glucosides, cyanidin 3-*O*-[2-*O*-(β-glucopyranosyl)-6-*O*-(trans-feruloyl)-β-glucopyranoside]-5-*O*-[6-*O*-(malonyl)-β-glucopyranoside] cyanidin 3-[2-(glucosyl)-6-(*cis*-*p*-coumaroyl)-glucoside]-5-[6-(malonyl)-glucoside]	[50,51]
		Red	Anthocyanins	Pelargonidin 3-sophoroside-5-glucoside, pelargonidin 3-[2-(glucosyl)-6-(*trans*-*p*-coumaroyl)-glucoside]-5-glucoside, pelargonidin 3-[2-(glucosyl)-6-(*trans*-feruloyl)-glucoside]-5-glucoside, pelargonidin 3-[2-(glucosyl)-6-(*trans*-*p*-coumaroyl)-glucoside]-5-(6-malonylglucoside), pelargonidin 3-[2-(glucosyl)-6-(*trans*-feruloyl)-glucoside]-5-(6-malonylglucoside), 3-*O*-[2-*O*-(b-d-glucopyranosyl)-6-*O*-(trans-caffeoyl)-b-d-glucopyr-anoside]-5-*O*-(6-*O*-malonyl-b-d-glucopyranoside), pelargonidin 3-*O*-[2-*O*-(b-d-glucopyranosyl)-6-*O*-(*cis*-*p*-cou-maroyl)-b-d-glucopyranoside]-5-*O*-(6-*O*-malonyl-b-d-glucopyranoside	[52,53]
Kohlrabi (*Brassica oleracea* var. *gongylodes*)	Swollen epicotyl	Purple	Cyanidin and cyanidin glucoside	Cyanidin-3-diglucoside-5-glucoside, cyanidin-3-(sinapoyl)-diglucoside-5-glucoside, cyanidin 3-(feruloyl) (sinapoyl) diglucoside-5-glucoside	[54,55,56,57]
Taro (*Colocasia esculenta*)	Corm	Purple		Cyanidin and pelargonidin glucosides	[58]
